# Dietary habits, body image, and health service access related to cardiovascular diseases in rural Zambia: A qualitative study

**DOI:** 10.1371/journal.pone.0212739

**Published:** 2019-02-22

**Authors:** Yukiko Tateyama, Patou Masika Musumari, Teeranee Techasrivichien, S. Pilar Suguimoto, Richard Zulu, Christopher Dube, Mitchell D. Feldman, Masako Ono-Kihara, Masahiro Kihara

**Affiliations:** 1 Department of Global Health and Socio-epidemiology, School of Public Health, Graduate School of Medicine, Kyoto University, Kyoto, Japan; 2 Medical Education Center, Graduate School of Medicine, Kyoto University, Kyoto, Japan; 3 Institute of Economic and Social Research, University of Zambia, Lusaka, Zambia; 4 Ndola District Health Office, Ndola, Zambia; 5 Department of Medicine, University of California San Francisco, San Francisco, California, United States of America; Florida International University Herbert Wertheim College of Medicine, UNITED STATES

## Abstract

**Background:**

Cardiovascular diseases are among the leading causes of mortality and morbidity in sub-Saharan Africa, including Zambia, where cardiovascular diseases account for 8% of the mortality rates. Despite an increasing number of cardiovascular disease-related studies in Zambia, qualitative studies exploring how cardiovascular diseases and their risk factors are understood in the socioeconomic and cultural contexts are still few. This study, therefore, aimed to analyze the beliefs, perceptions, and behaviors related to cardiovascular diseases and their risk factors among the local residents of Zambia.

**Methods:**

This qualitative study was conducted from August to September 2014 among healthy residents aged 40 years and above in a rural community in Mumbwa District. We investigated the beliefs, perceptions, and behaviors related to cardiovascular diseases and their potential risk factors in the sociocultural context of Zambia by conducting in-depth interviews and focus group interviews. Audio-recorded interviews were transcribed and analyzed using thematic analysis with investigator triangulation.

**Results:**

We conducted 34 in-depth interviews and 6 focus group interviews with 27 males and 40 females. Most participants were aware of the prevalence of cardiovascular diseases around them and correctly identified hypertension, excessive salt, sugar, and cooking oil intakes, poor quality cooking oil, consumption of meat or vegetables contaminated with chemicals, obesity, stress [“thinking too much”], lack of physical exercise, and heredity as potential risk factors of cardiovascular diseases, while smoking and alcohol were mentioned by only a few participants. However, they claimed that many of these risk factors were difficult to avoid due to ingrained taste preferences for high salt and sugar, increasingly busy lives that force them to use cooking oil to reduce preparation time, cultural preference for big body size or fatness, especially for women, stigmatized body image attached to HIV, stressful life or life events related to poverty, and financial barriers to affording quality foods and healthcare services. Limited health screening opportunities and the negative impact of HIV-related stigma on health-seeking behavior also emerged as important risk factors for cardiovascular diseases.

**Conclusions:**

This study revealed that participants are relatively well aware of cardiovascular diseases and their risk factors. However, they engage in high-risk health behaviors, due to ingrained taste preferences, limited knowledge, and unavoidable socioeconomic and cultural circumstances. Results suggest that prevention interventions addressing cardiovascular diseases in rural Zambia should target gaps in knowledge and socioeconomic and cultural barriers.

## Introduction

Non-communicable diseases (NCDs), mainly cardiovascular diseases (CVDs), cancer, chronic respiratory diseases, diabetes, and mental disorders, are now the leading causes of mortality and morbidity in low- and middle-income countries (LMIC) [1]. Of the total global mortality due to NCDs in 2012 (38 million), 80% occurred in LMIC [1]. Moreover, of the premature deaths (before age 70) attributable to NCDs, 37% were due to CVDs and 82% occurred in LMIC [1].

Globally, CVDs are strongly associated with a common set of behavioral risk factors, including tobacco use, unhealthy diet, physical inactivity, harmful use of alcohol, and obesity [1]. Poverty, stress, and heredity are also documented risk factors for CVDs [1]. In LMIC, although population aging due to demographic transition (shift of fertility and mortality from a high to a lower state) is contributing to some extent to the increased incidence of CVDs, unhealthy changes in lifestyle patterns due to globalization, increased industrialization, and urbanization are recognized as the most salient factors propelling the epidemic [2,3]. In sub-Saharan Africa (SSA), more than 2.06 million deaths due to NCDs were reported in 2010, which represents a 46% increase from 1990 [4]. There is a strong indication that the greatest increase in NCDs over the next decade will occur in the African and Eastern Mediterranean regions [2,3].

Zambia, like many other countries in the region [1,5], is witnessing a significant increase in the burden of NCDs and their risk factors [6] against the backdrop of infectious diseases (such as HIV, malaria, and tuberculosis), and reproductive, maternal, newborn, and childhood health disorders [7]. In 2012, 8% of the total deaths were due to CVDs in Zambia [1,8]. In a recent mixed-methods study in Zambia, participants linked hypertension to westernized diets, lack of physical activity, stress, and urbanization. In addition, it revealed a prevailing sense that hypertension was something beyond their control, as it was associated with “unavoidable circumstances of life” such as stress due to death or illness in the family [9].

Going forward, mitigation of the ongoing epidemic of CVDs will require sound evidence and an in-depth understanding of the risk factors for CVDs and the specific context in which they occur. While there are a growing number of epidemiological studies and vital statistics on the risk factors for CVDs in Zambia [9–20], qualitative studies exploring how CVDs and their risk factors can be understood in their socioeconomic and cultural contexts remain remarkably scarce [9]. An in-depth analysis of qualitative data may provide different perspectives on our understanding of CVDs and is likely to lead to more informed policymaking [21]. Therefore, the purpose of the current research was to explore the beliefs, perceptions, and behaviors related to CVDs and their risk factors among the local residents of Zambia. Mumbwa District, a rural area in the Central Province, was selected as the study site because it has begun to experience urbanization and economic growth, making it a good setting to understand the complex interactions between such social changes and traditional culture and values.

## Methods

### Participants and study setting

This study was conducted from August to September 2014 in Mumbwa District in the Central Province of Zambia. The district is located about 150 km west of the capital of Lusaka and is home to approximately 210,847 inhabitants [22]. For this study, we selected communities located near the Mumbwa Township clinic, the primary health center for Mumbwa residents. Self-reported healthy Mumbwa residents aged 40 years and above, both male and female, were invited to participate through word-of-mouth recruitment by one of the authors (Y.T.) and also by field assistants. Participants were recruited through purposive sampling such that the participants varied as widely as possible with respect to sex, age, educational level, economic status, and job status [21]. Recruitment of the participants was terminated when thematic saturation was attained [23].

### Data collection

We conducted in-depth interviews (IDIs) and focus group interviews (FGIs) using a semi-structured interview guide developed based on an extensive literature review on the subject of NCDs in SSA [2]. The interview guide explored various topics related to lifestyle (including dietary habits), cultural beliefs related to health, health-seeking behaviors, and the perceptions of lifestyle-related CVDs and health conditions, such as obesity, hypertension, and diabetes, that elevate the risk of CVDs.

The field research team included the first author (Y.T.) and field assistants who were fluent in the local languages and trained in the content and procedures of the study upon recruitment. Potential participants were provided with an explanation of the research objective, either in English or in their local language along with an information sheet. Only participants who granted informed consent were included in the study. Appropriate measures were established to assist participants who needed psychological support upon completion of the interviews. These included referrals to counseling services operated by local counselors.

The interview venue was selected based on the convenience and preferences of the participants, taking into consideration their privacy and comfort. Locations included participants’ houses, schools, and open spaces in the community. The demographic data of each participant were collected using a short structured questionnaire before the interview. The interviews were conducted in the language that was most suitable for the participants (English, Nyanja, Tonga, Bemba, or Lozi) and recorded using a digital voice recorder upon approval of the participants. Interviews conducted in the local languages were consecutively translated into English by the field assistants, and both the interviews in the local language and their English translations were audio-recorded to confirm the appropriateness of the translation. The interviews recorded in English were then transcribed by one of the local assistants, with the accuracy of transcription confirmed by the principal investigator (Y.T.). Field notes were taken to record the physical circumstances of the participants and research settings, including the foods at home and those available at shops and markets. The time spent on IDIs and FGIs was around 40–110 minutes.

### Analysis

The transcribed data were manually analyzed using a thematic analysis procedure with investigator triangulation, which involved independent coding and categorizing, followed by a series of discussions by 4 investigators to minimize the possibility of bias coming from any individual investigator (Y.T., P.M.M., T.T., and S.P.S.) and to improve the interpretation of the data and credibility of the analysis. The analytic approach involved (1) becoming familiar with the data through an iterative process of reading the dataset transcripts, (2) generating initial codes, (3) arranging the codes into larger categories, and (4) drawing connections between the codes and categories until a saturated thematic map of the analysis was generated [24,25]. Quotes from the participants were provided to support the themes.

### Declarations

This study was granted ethical approval from the Ethics Committee of the Graduate School and Faculty of Medicine of Kyoto University, Japan (No. 1097) and the University of Zambia Biomedical Research Ethics Committee, Zambia (No. 007-06-14). All participants provided written informed consent prior to participating in the study.

## Results

A total of 67 Mumbwa residents participated in this study through 34 IDIs and 6 FGIs (each FGI consisted of 3 to 7 participants). Forty participants (59.7%) were women, 31 (46.3%) were widows or widowers, and 19 (28.4%) were without regular employment; 26 participants (38.8%) had a monthly income of less than 500 Kwacha (US $100 dollars). All participants were Christian by religion. Twenty-two participants (32.8%) reported hypertension, while 4 (6.0%) reported diabetes, and 6 (9.0%) reported a history of stroke (Table 1).

**Table 1:** Characteristics of the participants.

**Characteristics**		**n = 67**	**%**
Sex	Male	27	40.3
	Female	40	59.7
Age	40s	25	37.3
	50s	17	25.4
	60s	15	22.4
	70s and older	10	14.9
Marital status	Single	3	4.5
	Married	28	41.8
	Divorced	5	7.5
	Widow/widower	31	46.3
Education level	None	11	16.4
	Primary	27	40.3
	Secondary	17	25.4
	More than college	12	17.9
Employment status	Employed	16	23.9
	Self-employed	32	47.8
	Unemployed	19	28.4
Monthly income (US Dollar)	Less than 100	26	38.8
	100–400	15	22.4
	> 400	12	17.9
	Unknown	14	20.9
Medical history (self-reported)	Hypertension	22	32.8
	Diabetes	4	6.0
	Stroke	6	9.0

The interviews and field observations revealed a variety of foods available for the local residents at the markets, shops, and street stalls, as classified in Table 2.

**Table 2:** Foods available at the markets, shops, and street stalls in the study area.

Vegetables	cabbages, carrots, *chibwabwa* (pumpkin leaves), Chinese cabbages, *delele* (okra), groundnuts, *impwa* (small white eggplants), *kalembula* (sweet potato leaves), mushrooms, onions, *rape* (green leafy vegetable), sweet potatoes, green peppers
Meat/Fish/Other protein products	beef, chicken, goat, pork, sausages, dried fish, fish, *kapenta* (small fish), eggs, beans, *soya pieces* (soy meat)
Staple food	bread, maize products [*nshima*, porridge, *sampo* (cooked maize mixed with groundnuts)], rice
Seasonings	butter, cooking oil, peanut butter, salt, sugar
Snacking	cream doughnuts, fritters, popcorn, scones
Drinks	soda drinks (e.g., Coca-Cola, Fanta), traditional Zambian beverages (e.g., *Maheu*), milk, tea
Fruits	apples, bananas, guavas, mangoes, oranges, watermelons

Local names are shown in italics.

The themes that emerged from the IDIs and FGIs are presented below as headings and supported by quotes from the participants. Each quote is accompanied by the sex and age of the participant in parentheses. In one of the FGIs, the participants included women only in their 40s. The age of these women is presented as “age in 40s” because it was often difficult to identify which participant was speaking in the voice recordings.

### Theme 1: Awareness about CVDs and hypertension as their cause

Overall, the participants seemed to be well aware of stroke and heart disease and the fact that hypertension is a major cause. They mentioned that these conditions were already common in their community and often led to death. These conditions were reported from many of our participants (13 individuals and 5 focus groups) as their own problems and also as the problems of individuals around them, such as siblings, relatives, friends, and neighbors. Hypertension was cited as a cause of stroke and heart disease by many of the participants (8 individuals) and was also noted to be increasing (5 individuals and 4 focus groups) because it had started occurring even among non-obese people, whereas it was previously only common among obese people.

*“People who got stroke are many*. *Even my neighbor and relatives passed away because of stroke*. *Hypertension is common*.*” (Male*, *age 72)*

*“At the moment*, *hypertension has no age*: *it can attack anyone at any time…” (Female*, *age in 40s)*

*“People who suffer from high blood pressure used to be very fat*, *but nowadays even people who are thin can suffer from hypertension*. *So*, *I have no idea*.*” (Female*, *age 70)*

Heredity was cited from some participants (3 individuals and 1 group) as a cause of hypertension:

*“BP (blood pressure) is maybe family inheritance*. *In the clinic*, *people are asked*, *‘Do you have someone who has high blood pressure in your family*?*’ If they say yes*, *they are told that it is because of inheritance like family disease*.*” (Female*, *age 42)*

### Theme 2: Perceived life-related risk factors or causes of CVDs

A majority of the participants (20 individuals and 3 focus groups) in this study reported excessive consumption of salt, sugar, and cooking oil, although they mentioned that they were mostly aware that this was unhealthy behavior. They reported that the inclination towards such dietary habits was inherent to food and taste preferences that they and their family members had developed at home or at school throughout their lives. The net consumption of cooking oil appeared to be particularly high because many participants (17 individuals) reported the frequent use of cooking oil in large amounts. We observed that many families consumed the oil almost to the last drop instead of discarding it. Lifestyle changes also appeared to be promoting the excessive consumption of cooking oil. Two participants reported that using cooking oil enabled them to save time amid their busy schedules. Consequently, the use of excessive cooking oil became an important dietary habit to expedite cooking.

<Salt>

*“I do use salt*, *and most of my family members like salt*. *I know eating too much salt is bad… (But) No*. *I can’t (stop using salt a lot)*. *I don’t know why … just for taste*.*” (Female*, *age 40)*

<Sugar>

*“There is this “Zigolo” (referring to the mixture of sugar and water)*, *when we were at school*, *we mixed sugar and water until the sugar could no longer dissolve*. *This was believed to be the best mixture*. *So we become used to that kind of life at school and continue doing it at home*. *I think this behavior is stuck in my head*, *so I cannot take just a little bit of sugar*.*” (Male*, *age 44)*

*“People use A LOT of sugar*. *Some eat sugar*, *even adult*. *… It’s an abuse* … *People don't know what can happen by taking overloaded sugar*. *They don't know the danger*.*” (Male*, *age 58)*

<Cooking oil>

*“If you go deep in the village*, *a 2*.*5-liter bottle of cooking oil may take a month to finish*. *But among people like us (people in the township)*, *it will take less than a week to finish*.*” (Female*, *age in 40s)*

*“Most times we are found at our workplace*. *It (cooking oil) is just a cooking shortcut to save time*.*” (Female*, *age in 40s)*

Many participants also pointed out recent changes in the quality of foods available at the market that were potentially harmful and could lead to obesity, hypertension, heart disease, and stroke. Many of the participants (14 individuals and 4 focus groups) expressed their concerns regarding “chemicals”, such as the fertilizers put on the vegetables or the growth-promoting agents used in animal products. Others (12 individuals) also questioned the quality of the products they had recently consumed, especially cooking oil, which used to be liquid but now was in most cases solidified. Although they were concerned that cheaper solidified cooking oil could affect their health, they were unable to avoid it due to financial barriers.

<Chemicals>

*“A long time ago*, *we never used the chemicals that are being used nowadays* … *These days*, *we cannot grow any foods without using any chemicals* … *We can develop stomachache and heart problems (from chemicals)* … *Chemicals like fertilizers bring problems*.*” (Female*, *age 84)*

*“For example*, *look at the broiler chickens*. *They are injected with chemicals that make them grow very fast*. *If you eat this chicken*, *you will also be affected by the chemicals that are found in the chicken*. *This contributes to being fat*.*” (Male*, *age 47)*

<Thick cooking oil>

*“If you buy a five-liter container of cooking oil*, *when you buy it*, *it is fine*, *but later it becomes thick*. *If we eat solidified cooking oil*, *it is going to be the same in my veins*. *So I think it is dangerous …” (Male*, *age 44)*

*“*… *There is thick cooking oil on the market that is common now*. *The people who manufacture cooking oil don't care about people but the money only*. *It is not good for our health*. *We can develop blood pressure*, *hypertension*, *and heart attack*.*” (Female*, *age 63)*

*“We used to use light cooking oil*, *but nowadays it is thick*. *But what we can do is just to buy the cheapest cooking oil due to financial problems*. *I don’t know that thick cooking oil brings something (bad) in my body or not*, *just I have to buy*.*” (Female*, *age 78)*

In this study, daily life stressors mostly due to poverty or traumatic life events, such as the loss of family members, were cited as the main factors contributing to hypertension. Many participants (16 individuals and 6 focus groups) frequently mentioned that “thinking too much” to cope with the day-to-day poverty and hardships of life could lead to hypertension. This belief was reinforced by the advice they received from their healthcare providers.

<Stress>

*“I can say poverty is contributing because there are a lot of people dying*. *In our society*, *we have to take care of others and it causes us pressure*. *This pressure will make us start ‘thinking too much*,*’ which can cause high blood pressure*.*” (Female*, *age in 40s)*

*“I was told at the hospital that ‘thinking too much’ can cause high blood pressure*. *Because when I think too much*, *my BP (blood pressure) goes up*. *I think about my husband*, *my children who died*. *Even I lost my grandchildren*. *Thinking too much and sorrow contribute to my high blood pressure*.*” (Female*, *age 78)*

In contrast to the types of behaviors discussed above, tobacco smoking and drinking were not common among the participants we studied. Only 2 individuals cited smoking as a risk factor for heart disease, but not for stroke or hypertension. Smoking was more frequently associated with diseases such as cancer, tuberculosis, and asthma, while alcohol consumption was more frequently associated with poverty and the risk of contracting HIV or other sexually transmitted diseases (STDs).

<Smoking>

*“I used to smoke but now I do not smoke because it is affecting my heart*. *My heart span will be reduced if I continue smoking … It (drinking) can affect your heart*, *it can bring diseases*.*” (Male*, *age 64)*

*“It is bad to smoke because someone will develop cancer*, *tuberculosis*, *asthma as well*. *Tuberculosis is coming from that smoke*.*” (Female*, *age 42)*

<Drinking>

*“It (alcohol) brings poverty*. *People spend the time only for drinking*, *and not doing anything*.*” (Female*, *age 40)*

*“… It is good to drink alcohol*. *But if you overdrink*, *you lose your senses and involve yourself in sexual activities and end up contracting HIV*, *STDs*.*” (Female*, *age 53)*

### Theme 3: Beliefs and perception about fatness

Some of the participants (5 individuals) reported that “being fat” is an accepted norm in their society. However, they also mentioned that fatness could lead to hypertension and diabetes if it became “abnormal.”

*“They think people (who are overweight) are good because they think they eat very well*. *…*.. *They consider fat people to be very wealthy*.*” (Female*, *age 84)*

*“Generally*, *people may like fatness*, *but they should control their weight … (We need to be) just normal size*, *not to be abnormal*. *When becoming abnormal*, *it will be a big problem*, *and those people will just collapse because of the fatness*. *Even hypertension and diabetes also will come*.*” (Female*, *age 53)*

Interestingly, there seems to be a difference in the cultural significance associated with “being fat” between men and women. One individual stated that female fatness was better accepted than male fatness, because the latter was regarded as a sign of laziness. In contrast, thinness was associated with “being lazy” among females.

*“For men*, *just an average body is better in the tradition*. *If the men are fat it means that he is lazy*, *and getting too fat means that he cannot do anything*. *But for women*, *wife is fat means that her husband knows how to keep her and his family*, *so it is no problem*.*” (Female*, *age 42)*

*“In our tradition*, *some people say that* … *if you marry a slim lady*, *she will be lazy and nothing to do any housework*, *but a big lady will do anything for you*. *(So) fatter is better than slim*. *People prefer fatter lady*, *because she will be working well*.*” (Female*, *age 42)*

In this study, a considerable stigma attached to body image in the context of the HIV epidemic was noted. Two individuals associated thinness or weight loss with HIV infection. However, in a few instances, obesity was conversely associated with HIV and the initiation of antiretroviral therapy (ART), with the belief that ART leads to increased appetite, thereby resulting in weight gain.

*“HIV/AIDS also contributes to becoming fat*, *because if you lose weight people will think you are HIV-positive.” (Female, age in 40s)*

*“Look at people that have HIV/AIDS, they become fat after starting the medicine (ART). I don’t know whether medicine boosts their body or just eating a lot*. *… some HIV (patients) get very fat and develop asthma or high blood pressure if people become fat. So those are also problems we face.” (Female, age 42)*

### Theme 4: Beliefs about the prevention of CVDs

There were divided opinions among the participants as to whether stroke or hypertension was preventable. Some of the participants (4 individuals) believed that stroke or hypertension could be prevented through appropriate diet and physical exercise, while others (2 individuals) considered them unavoidable:

*“We can prevent this kind of disease by eating the right food that will not affect your health or your body*.*” (Female*, *age 63)*

*“… Exercising also helps prevent such diseases like blood pressure and stroke*. *I had the same disease because of lack of exercise*.*” (Male*, *age 47)*

*“For HIV/AIDS*, *you can protect yourself by behaving in a proper manner*. *But diseases like stroke and blood pressure—you cannot avoid them*.*” (Female*, *age 40)*

*“People do not care about blood pressure*. *They think BP (blood pressure) and stroke are a natural disease*. *So people don’t care about what they eat*.*” (Male*, *age 47)*

### Theme 5: Access to health check-ups and its determinants

In the community we studied, no regular health check-up program for CVDs was available. Typically, individuals were incidentally diagnosed with hypertension when they attended a health facility for medical conditions other than CVDs, such as common infectious diseases. Some participants (1 individual and 1 focus group) further stated that the health facilities do not provide effective preventive services, giving priority to care and treatment rather than to preventive medical check-ups or screening:

*“When I developed some illnesses and was taken to the hospital*, *I was diagnosed with high BP (blood pressure) as well*.*” (Female*, *age 54)*

*“In Zambia*, *if you go to the hospital when you are not sick*, *no one will attend to you*. *…*. *Even if medical check-ups are supposed to be performed there*, *no one will provide the service*.*” (Female*, *age in 40s)*

There were a few accounts in which the fear of disclosure of HIV was reported as a factor that discouraged visits to health facilities. Most health facilities in the community offered provider-initiated HIV testing and counseling, which tended to negatively impact people in the process of diagnosis and treatment of diseases other than HIV/AIDS, due to the social stigma attached to HIV. In addition, the participants’ health-seeking behaviors and health service utilization activities existed in competition with other pressing needs, such as finding work and having enough to eat. Such circumstances appeared to be common among our participants since most of them (51 individuals) answered that they had unstable employment in the pre-interview questionnaire. In addition, 1 individual expressed concern over the prospect that she might not be able to continue antihypertensive medications in the future due to the financial costs or drug shortages in healthcare facilities.

*“I have seen three friends who were HIV-positive and (were) attacked (by stroke)*. *Because they didn't disclose their HIV status*, *it became stress for them* … *They thought that if they go to the clinic*, *their blood will be checked*, *and then they (medical workers) will know their (HIV) status*. *So they keep their health conditions secret*, *inside their hearts …” (Female*, *age 42)*

*“I have to look for money and food*, *so I do not usually find time to go to the hospital*. *I need to find food to eat*.*” (Female*, *age 78)*

*“I should only take the medicine when my blood pressure spikes*. *I was encouraged to control myself because in the future I might not be able to get the medicine*, *and eventually I might even die*.*” (Female*, *age 67)*

## Discussion

This is one of the first qualitative studies to explore the beliefs, perceptions, and behaviors related to CVD risk factors in the socioeconomic and cultural contexts of Zambia. The participants were aware that CVDs are increasing and are common in their community based on their personal experiences or those of acquaintances who suffered from CVDs or hypertension. The participants were also able to identify a number of risk factors associated with CVDs. However, their knowledge was still limited, as documented in studies conducted in other SSA countries [26]. In addition, knowledge did not translate into healthy behavioral practices owing to the socioeconomic, cultural, and structural factors that affect their dietary habits, cooking styles, body image, and beliefs regarding prevention and that also create stressful life circumstances and limit their access to quality foods, healthcare services, and health screening programs.

### Socioeconomic impacts on dietary habits and food quality

The participants were well aware of the relationship between unhealthy diets and CVDs. However, among them, engaging in healthy dietary habits competed not only with individual-level and socioculturally driven food tastes and preferences, but also with complex forces well beyond the control of individuals, namely the uncontrolled growth of the market as well as the financial barriers that constrain their access to quality foods. Excessive intakes of salt, sugar, and cooking oil were reported by most of the participants as dictated by food and taste preferences nurtured at home and/or at school. Busier lifestyles also appeared to be contributing to the use of cooking oil to save time, thereby fostering a preference for oily foods. Evidence indicates that food and taste preferences play a significant role in the dietary behaviors of individuals [27]. However, interventional studies promoting healthy dietary habits that are sensitive to the existing tastes and food preferences are extremely rare in SSA [28]. Therefore, there is a pressing need for more focused research in this area to test the effects of health education and/or interventions that facilitate appropriate personal risk assessments for CVDs and changes in the social norms of food and taste preferences.

The perception that an unhealthy diet was associated with the effects of uncontrolled market growth driven by globalization on the types and quality of foods available at the market was also documented in previous research in Zambia [9]. The participants in the present study had strong beliefs that the foods sold at the market were of poor quality and were responsible for the increased risk of CVDs. The participants blamed the use of fertilizers, growth-promoting agents in animal products, and low-quality cooking oil. In parallel, financial insecurity not only limited the participants' ability to access quality foods (through decreased purchasing power or by creating other competing needs), but it also interfered with their health-seeking behavior and healthcare service utilization. Financial barriers and globalization-related factors are structural elements that will require systemic actions through policies promoting the availability of and access to healthy and nutritious diets.

### Sociocultural perception of fatness

Overweight/obesity has been well documented as a major and modifiable risk factor for CVDs [1], and our study participants were largely aware of this association. However, being fat was a socioculturally desirable status in the setting of our study. Overall, fatness was framed as a symbol of wealth, an observation also reported in previous research [29,30]. In addition, there was a gender disparity, whereby female fatness was viewed more favorably compared to male fatness. This cultural perception, along with others documented elsewhere (e.g., personal values and preferences and the stigma attached to thinness) [2,31,32], could partly explain the higher prevalence of overweight/obesity among females compared to males in SSA. For example, in West Africa, the increased prevalence of obesity over the past decade was almost entirely among women [33]. Moreover, in a previous study in South Africa, approximately half of the sampled women but none of their male counterparts were obese or overweight [34]. In parallel, Zambia has one of the highest prevalence rates of HIV in the region, which was 13% in 2013–2014 [35], and the stigma associated with this disease also influences social perceptions of body size. In our study and studies elsewhere [29,36,37], thinness and/or weight loss was associated with HIV/AIDS. The cultural desirability of fatness, gender disparity in the acceptance of fatness, and HIV-related stigma attached to weight loss (that is, if people lose weight, they will be perceived to be HIV-positive) all pose serious barriers to weight control in Zambia and should be addressed appropriately through interventions aiming to shift social norms and perceptions of body size.

### Life-related hardships and stress: Impact on the belief of prevention

In line with the results of other studies [9,38,39], we found that psychological stress, expressed as “thinking too much,” was a significant factor associated with CVDs and hypertension by the participants. The psychological stress was mainly due to the financial hardship of day-by-day life or traumatic life events such as the loss of family members. We also found that the participants frequently mentioned that nothing could be done to control the occurrence of CVDs. This perception seemed to be nurtured at least in part by the lack of knowledge as well as by the beliefs that the psychological stress and other traumatic life events contributing to CVDs were unavoidable circumstances. Similar results have been reported in previous studies from Zambia [9] and Cameroon [33]. In our study, the perception that “thinking too much” could contribute to hypertension and CVDs often derived from the advice that participants received from their healthcare providers. While the potential mechanisms of how stress leads to adverse health outcomes have been well established by theories such as *allostatic load* [40], such information could be dangerous if it is misinterpreted to mean that the impact of “thinking too much” outweighs that of other modifiable factors, thereby depriving individuals of motivation to engage in healthier behaviors. This perception of “unavoidability” should be carefully addressed through community-based health education programs targeting communities as well as healthcare providers, for example.

### Social and structural barriers to healthcare services

Consistent with other studies [2,5,41], our participants perceived that the healthcare system was more oriented toward treatment and care than toward prevention. This in turn affected their health-seeking behavior by inhibiting their screening opportunities for NCDs. The influence of the HIV epidemic and its stigmatization on health-seeking behavior and healthcare service utilization was another important finding in this study. The fear of knowing their HIV status prevented individuals from attending health facilities where provider-initiated HIV testing and counseling were being implemented. This could be regarded as an unintended consequence of the HIV testing programs widely implemented in Zambia [42]. This finding highlights the urgent need for interventions that properly prioritize and harmonize prevention and treatment programs for HIV and CVDs within the communities of Zambia.

CVD occurrence has rapidly increased in the last decades and now accounts for a substantial proportion of the disease burden in SSA. Mortality due to CVDs increased by 81% from 1990 to 2013 [43], and a growing body of evidence predicts a significant increase in the incidence of CVDs over the next decade in this region [44,45]. Despite the implementation of initiatives and measures to tackle CVDs in many developed settings [46], such initiatives remain unacceptably scarce in many developing regions, particularly in SSA [47,48]. Our study provides further insights into the perceived association of various factors with the increased risk of CVDs in SSA at large and in Zambia in particular [2,49,50]. The findings of our study may inform interventions aiming to decrease the risk of NCDs in rural Zambia by suggesting that such interventions should address sociocultural and structural factors, including healthcare services, to increase their potential effectiveness. Finally, only a few participants had smoking or drinking habits and rarely related these habits to CVDs, which was likely due to a sampling bias and/or a lack of knowledge among participants. However, given the strong possibility of a future increase in these behaviors due to the pressures of market globalization, the development and implementation of appropriate health education programs that highlight alcohol and tobacco consumption as risk factors for CVDs and other NCDs are critical before both habits become highly prevalent in this community and too entrenched to change.

### Limitations

The possible generalizations from this study are limited by the sampling method as well as by the qualitative nature of the study that is based on subjective beliefs, perceptions, and opinions. The results may not accurately represent the CVD risk factors throughout rural Zambia, as this was a qualitative study that was conducted using semi-structured interviews with a small non-random sample. It is clear that a more controlled, quantitative study is needed to test the perceived associations reported in this study. Nonetheless, the number of participants was adequate to permit qualitative analysis and data saturation, considering that the participants varied widely in terms of sex, age, and education level.

## Conclusion

This study helped to elucidate the beliefs, perceptions, and behaviors associated with CVDs and their risk factors. The results suggest that interventions addressing the risk factors of CVDs in rural Zambia should target not only the sociocultural factors affecting food taste and preferences, cultural preference of overweight/obesity, and HIV-related stigma attached to thinness, but also the structural barriers such as financial insecurity, effects of market globalization, and health services accessibility. Individualized health education interventions should be encouraged to challenge misconceptions such as the inevitability of the development of CVDs.
